# Dr. Leverson and the Bugaboo of Small-pox

**Published:** 1897-05

**Authors:** Robert N. Taylor

**Affiliations:** “Pure” Homeopathic Layman; Office of Taylor & Agard, No. 35 S. Front Street, Philadelphia


### DR. LEVERSON AND THE BUGABOO OF SMALL-POX.

Office of Taylor & Agard, No. 35 S. Front Street,
Philadelphia, April 13th, 1897.

Editor of The Homeopathic Physician.

Sir:—In the March number of your journal, at page 108, in commenting on Dr. Leverson's article, entitled, "The Bugaboo of Small-pox," you express your surprise at his statement that small-pox is a mild disease, and can be cured in a few days. No doubt Dr. Leverson made the statement on the authority of Dr. Hadwen, of Gloucester, England, who, in a recent public address, gave investigated statistics of two so-called "quacks," one who treated by hydropathy, the other by the oil treatment—solidified oil. Of 260 cases investigated by Dr. Hadwen, treated by the latter treatment, only four died, about two per cent., while the cases treated in the pest-house, under the supervision of the local Board of Health, showed fifty-four per cent. of fatalities. In the same connection I would like to ask if you have ever given an expert opinion on Dr. Leverson's pathological diagnostic table, which shows that cow-pox virus does not produce a single similar symptom to small-pox, and which, if true, shows that no homeopathic physician has any right to vaccinate under any circumstances.

ROBERT N. TAYLOR,
*"Pure" Homeopathic Layman.*

